# Spontaneous Coronary Artery Dissections: A Systematic Review

**DOI:** 10.3390/jcm10245925

**Published:** 2021-12-17

**Authors:** Giovanni Teruzzi, Giulia Santagostino Baldi, Sebastiano Gili, Gianluca Guarnieri, Piero Montorsi, Daniela Trabattoni

**Affiliations:** 1 Centro Cardiologico Monzino, IRCCS, Invasive Cardiology Department, 20138 Milan, Italy; giovanni.teruzzi@ccfm.it (G.T.); giulia.santagostino@ccfm.it (G.S.B.); Sebastiano.gili@ccfm.it (S.G.); gianluca.guarnieri@unimi.it (G.G.); piero.montorsi@ccfm.it (P.M.); 2 Department of Clinical Sciences and Community Health, University of Milan, 20122 Milan, Italy

**Keywords:** spontaneous coronary dissection, SCAD, clinical outcomes

## Abstract

Myocardial infarction with nonobstructive coronary artery disease due to spontaneous coronary artery dissection (SCAD) accounts for 5–8% of acute coronary syndrome (ACS) presentations. The demographic characteristics, risk factors, and management of patients with SCAD differ from those with atherosclerotic disease. The objective of this review is to provide a contemporary understanding of the epidemiology, pathophysiology, clinical presentation, and management of SCAD.

## 1. Introduction

### 1.1. Rationale

Spontaneous coronary artery dissection is an unpredictable, non-traumatic and non-iatrogenic separation of the coronary arterial wall. Despite being an uncommon cause of acute coronary syndrome, affecting young/middle-aged women in 85–90% of cases in published contemporary datasets, it is not rare. Indeed, the increased use of early angiography to assess acute chest pain presentations has resulted in recognition of spontaneous coronary artery dissection as more common. The condition poses diagnostic challenges and significant therapeutic dilemmas given the lack of research to guide management.

### 1.2. Objectives

Several registries and retrospective analyses have been performed on clinical presentation, incidence and recurrence of SCAD, angiographic characteristics and in-hospital and long-term clinical outcomes. However, no enough data exist regarding the ideal management of SCAD due to lack of randomized trials comparing medical therapy and revascularization strategies. Observations collected from contemporary SCAD case series have led to the general consensus that conservative therapy should be considered as a first-line approach in the absence of clinical high-risk features. In our review, we describe the pathophysiology, types of SCAD, risk factors, clinical presentation, and management approach with the aim of raising awareness of SCAD to facilitate accurate diagnosis promptly.

## 2. Materials and Methods

A systematic review was performed in accordance with the Preferred Reporting Items for Systematic Reviews and Meta-Analyses guidelines. We searched MEDLINE, Cochrane, Web of Science and Google Scholar databases. We used the following MeSH terms: mortality, death, survival, clinical outcomes. We used the following key words: survival, spontaneous coronary artery dissection, and SCAD, medical therapy. We included studies and recent review manuscripts.

### Search Strategy

A search strategy with free and controlled terms about spontaneous coronary artery dissection was established for the full search strategy with detailed database information accessed and peer review assessment.

## 3. Results

### 3.1. Epidemiology

Spontaneous coronary artery dissection (SCAD) is a non-atherosclerotic and non-traumatic cause of acute coronary syndrome and of cardiac death, in a few cases. It affects predominantly middle-aged (44–53 year-old) women, representing approximately 90% [1,2,3] of affected patients [2,4], despite having been reported during all lifespan.

Spontaneous coronary artery dissection is responsible for <1% of all acute myocardial infarction (AMI) [2,3,5,6], but its percentage increases dramatically (up to 25–30%) in women with AMI younger than 50-years of age [7,8] and is better diagnosed when coronary angiography is performed. In addition, SCAD accounts for 15–20% of AMI during pregnancy and the peripartum period [9].

### 3.2. Pathogenesis

SCAD is an acute coronary event due to the disruption of an epicardial coronary artery’s layers, due to intramural hematoma within the tunica media, which is not necessarily associated with an intimal tear, leading to separation of the intima from the remaining vessel and determining stenosis of the lumen and a subsequent acute coronary syndrome [10].

Two main pathophysiologic mechanisms supporting SCAD evolution have been proposed. The first one is the “inside-out” [11] hypothesis, where blood flows into the medial layer through an intimal tear creating a false lumen, which can propagate due to the intramural pressure [12,13]. On the other hand, according to the “outside-in” hypothesis, bleeding from a vasa vasorum creates an intramural haemorrhage and hematoma, without an intimal tear. More recently, the use of intravascular imaging, especially optical coherence tomography (OCT), allowed to determine that the second mechanism is the most frequent, showing that the false lumen is pressurized and that fenestration may arise from rupture of the false lumen into the true lumen [14].

In any case, the final result is a compression of the true lumen, determining an obstruction to flow and consequently myocardial infarction [3,5].

Pathological specimens showed infiltration of inflammatory cells, especially eosinophils, in the adventitia and periadventitial tissue, sparing the intima and media [15,16]. Intimal and medial sparing allows differentiating this condition from inflammatory artery diseases.

Typical risk factors are less represented in this patient population compared to myocardial infarction due to atherosclerotic plaque rupture; it is more likely influenced by sex, hormones, underlying arteriopathies, genetics, and physical and emotional stress [5].

The striking prevalence of SCAD in women leads to the hypothesis that hormones may play a role in the pathogenesis. However, to counter this hypothesis, both pre- and post-menopausal, nulliparous, postpartum, and multiparous women are affected in equal percentages [2,4,17] and SCAD affects both nulliparous and multiparous women. Conversely, it is unknown whether the absolute hormones level fluctuation is more relevant.

In addition to sex hormones, stress, either physical or emotional, has been reported as a trigger to SCAD; while emotional stress is more frequent in women, physical stress is more frequent in men [2,18,19,20].

### 3.3. Pregnancy-Associated SCAD (P-SCAD)

P-SCAD can occur at any time during pregnancy or in the post-partum period, being more frequent in the first week post-partum [21]. P-SCAD represents 5–17% of all SCAD cases [2,22,23] and 14.5–43% of AMI related to pregnancy [24,25]. The incidence is 1.81 per 100,000 pregnancies [9]. In addition, pregnant women with P-SCAD tend to be older at first childbirth and multigravidas.

In comparison to SCAD non-pregnancy related, P-SCAD has a more severe clinical presentation with impaired left ventricular function and shock, and more often left main and multivessel dissections occur [21,26,27,28].

Furthermore, approximately 15% only of SCAD occur in the peripartum period [2,22], with a more severe clinical course: ST-segment elevation myocardial infarction (STEMI) is the more frequently observed presentation, as well as an increased prevalence of left main involvement and a reduced left ventricular ejection fraction [21,27,29].

### 3.4. Clinical Presentation

Clinical presentation is similar to that of atherosclerotic coronary artery disease, with the vast majority of SCAD (>90%) [2,22,30] presenting with ST-elevation myocardial infarction in 20–50% of cases. In a minority of patients, ventricular arrhythmias (5%) [17,30,31] and cardiogenic shock (approximately 2%) may be the SCAD’s first clinical manifestation. The most striking difference from atherosclerotic coronary disease is the type of patient. Therefore, a high level of suspicion must be kept, in order to prevent delayed, or, even worse, missed diagnosis.

Classic symptoms include chest pain variably radiated to the arms, back, or jaw; dyspnoea; palpitations. EKG may demonstrate ST-segment myocardial infarction of being silent.

### 3.5. Diagnosis

**Figure 1:**
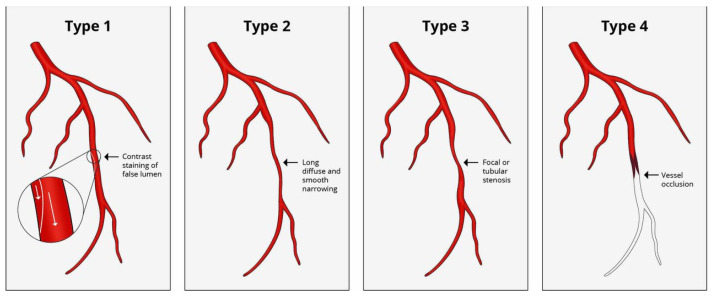
Angiographic spontaneous coronary artery dissection classification proposed by Saw et al. [17].

Coronary angiography is the main tool to diagnose SCAD and to define the extent of coronary involvement. Indeed, the identification of high-risk features warrants urgent revascularization. SCAD is classified into different categories based on angiographic appearance and the presence of intimal tear (Figure 1) according to the classification of Saw et al. [17]:

− Type 1: multiple radiolucent lumens due to intimal tear where contrast penetrate are visible. At times, a radiolucent flap is also visible. It can also show a stain of contrast dye within the false lumen [17]. The intimal tear separating true and false lumen is visible at the OCT imaging.
− Type 2: appears as a long vessel segment diffusely narrowed and tapering distally because of the intimal hematoma. These lesions are long and often show an abrupt change of caliper of the vessel diameter, either extending to distality or reacquiring normal caliper in the distality. Type 2A has a normal vessel at its extremities, while type 2B prolongs up to the distal part of the vessel. This type is the most frequent. OCT imaging shows a compressing intramural hematoma.
− Type 3: is similar to type 2, but shorter (<20 mm in length), so that it can mimic an atherosclerotic disease. It often requires intravascular imaging for the diagnosis. It is the rarest.
− Type 4: causes the occlusion of the vessel, mimicking coronary embolism.

SCAD can occur in any coronary artery, but the left anterior descending [22,32] is the most frequently involved coronary vessel [2,17]. Multivessel involvement is also frequent, being prevalent in 10–15% of cases [2]. In addition, the mid-distal segment of the vessel is the most frequently involved. Notably, isolated intramural hematoma has a worse prognosis compared to intimal dissection when treated conservatively [32].

Since the diagnosis is not always obvious with coronary angiography, intravascular imaging can confirm the diagnosis and exclude other causes of myocardial infarction, namely atherosclerotic plaque rupture. Unfavourable anatomy (i.e., severe tortuosity, false lumen wiring, distal involvement) should be considered a limitation to a safely intravascular imaging performance in SCAD patients. Indeed, procedural complications (i.e., extension of the dissection, flow impairment, and false lumen cannulation) occur in up to 8% of cases [14], even when intracoronary imaging is not performed. While OCT images are mostly diagnostic, IVUS images require careful attention to diagnose SCAD versus disrupted plaque, due to the lower spatial resolution of IVUS. Therefore, intravascular imaging is reserved only for cases where the diagnosis is uncertain.

If diagnostic uncertainty persists, after coronary angiography, coronary computed tomographic angiography (CCTA) or cardiac magnetic resonance (CMR) can be performed. CCTA is a non-invasive tool that can help in the diagnosis in uncertain cases because it allows visualization of dissection flaps and intramural hematoma and to assess healing. However, the spatial resolution of CCTA is suboptimal for small vessels, leading to false negatives, and soft atherosclerotic plaques can be mistaken for intramural hematoma [33]. Indeed, in the acute phase, <15% of dissections are identified, while better accuracy is demonstrated for sleeve-like hematomas and abrupt luminal changes [33,34] CMR showing late gadolinium enhancement in the territory of a suspected dissected coronary artery helps confirm the diagnosis, but a normal CMR does not exclude the diagnosis [28].

We report here our experience from a public service healthcare in Milan. Between January 2007 and November 2021, 6295 acute coronary syndromes (ACS) were diagnosed and treated with primary PCI, and 54 (0.8%) only were ACS SCAD-related (Table 1).

These data are comparable to those published in the literature (Table 2), as well as clinical presentation and type of treatment adopted. It is well known that SCAD mainly affects women and this observation is consistent among studies, however, our patients were older as we do not treat SCAD patients during their pregnancy period as well as after labor.

### 3.6. Treatment Strategy

The goal of therapy in SCAD is to maintain or restore cardiac function by improving coronary blood flow in the dissected artery.

Notably, treatment for P-SCAD is analogous to that of non-pregnancy-related SCAD, with caution to both maternal and foetal outcomes. Foetal radiation concerns suggested avoiding coronary angiography in stable pregnant women [27] but, due to higher mortality in pregnant women and to the negligible foetal radiation exposure with shielding, the standard of care treatment of AMI should be applied also to pregnant women [45].

#### 3.6.1. Interventional Management

The decision to proceed with percutaneous coronary angiography is based on clinical and anatomical characteristics.

Clinical features include clinical status, persistent chest pain with ongoing or persistent ischemia, hemodynamic instability, or ventricular arrhythmias, while high-risk procedural features are multivessel disease with proximal segments involvement, left main dissection, and distal flow, described by Thrombolysis in Myocardial Infarction (TIMI) grade [46,47,48]. Some authors suggest that adequate distal flow (i.e., TIMI 2–3) does not require PCI [46].

There are three differences in PCI in SCAD and atherosclerotic disease: 1. the pathological mechanism of SCAD is a medial dissection, compared to atherosclerotic plaque rupture; 2. PCI for SCAD is associated with worse outcomes; 3. most medically treated coronary dissections heal over time [35,36,49] Therefore, more than 80% of patients can be medically treated only [2].

Importantly, recurrent coronary dissections usually occur in different vessels. Therefore, treating one vessel does not prevent SCAD recurrences [35].

PCI in SCAD poses specific challenges: correct wiring of the true lumen may be challenging, long stent segments may be warranted to restore proper vessel flow and intramural hematoma may propagate downwards or backward, further impairing TIMI flow.

So far, the percutaneous revascularization goal should be an effective vessel reperfusion, after ensuring, both by angio and complementary intracoronary imaging (i.e., IVUS, OCT), that the guidewire is in the true distal lumen. An accidental false lumen wiring requires intimal fenestration with adequate dilators (i.e., cutting balloon), which is however considered a bail-out intervention.

Additionally, PCI success rate in SCAD is lower than that observed in PCI for atherosclerotic disease treatment (i.e., 47–72% in large cohort studies) [2,30,35]. PCI’s long-term adverse effect may also occur, including a late-acquired stent malapposition due to positive remodelling of the healed vessel. Recently few case reports and a small case series [50] have been published highlighting the possible advantages of Bioresorbable Vascular Scaffolds (BVS) in SCAD, as an option to a temporary scaffolding and the potential to a preserved physiology vessel restoration.

Coronary artery bypass grafting (CABG) for SCAD is technically feasible, but it is rarely used (less than 1% of SCAD are referred to CABG) [2] and limited to high-risk anatomic settings such as multivessel proximal dissection, left main involvement, or after PCI failure. CABG’s short-term technical and clinical success is high; however, on long-term graft patency it is definitely low due to native vessels’ competitive flow restoration after spontaneous vessel healing, leading to bypassing of the graft occlusion.

Selected case reports demonstrated the use of mechanical circulatory support as a bridge to recovery or to cardiac transplantation [51,52].

#### 3.6.2. Medical Therapy

In addition to MI management, treatment must be address chest pain resolution, prevention of recurrences and extra-cardiac abnormalities detection.

##### Antiplatelet and Anticoagulation Therapy

**Figure 2:**
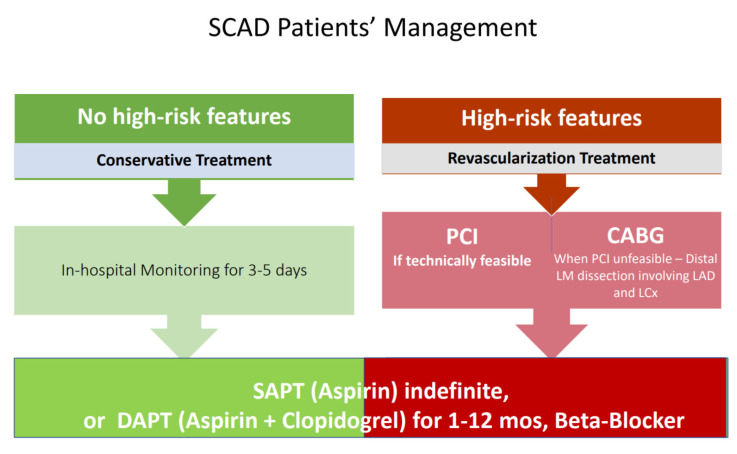
SCAD Treatment Algorythm. Figure legend: PCI: Percutaneous Coronary Intervention; CABG: Coronary Artery Bypass-Graft; SAPT: Single Antiplatelet Therapy; DAPT: Dual Antiplatelet Therapy.

Most patients diagnosed with SCAD receive at least one antiplatelet drug. However, no definite indications have been standardized regarding single or double antiplatelet treatment after SCAD. Indeed, while DAPT is warranted for patients receiving coronary stent implantation, expert consensus suggests that DAPT may be considered in the acute phase of SCAD and up to 12 months even in medically treated SCAD patients [46,47]. Over one year, antiplatelet therapy continuation should be assessed in a personalized manner (Figure 2).

The use of anticoagulation therapy balancing the prevention of intravascular thrombus formation in a vessel with slow flow vs. the risk of dissection propagation due to further intramural bleeding has to be considered. Therefore, an expert consensus document suggests that anticoagulation should be interrupted after a diagnosis of SCAD is posed [47].

In addition, thrombolysis is contraindicated in patients with SCAD due to the association of thrombolysis and clinical deterioration in this patient group [46].

##### Beta-Blockers, ACE Inhibitors and ARB

Beta-blockers, ACE inhibitors and ARBs should be prescribed according to MI and heart failure guidelines.

Beta-blockers may be particularly beneficial in this group of patients: in a study by Saw et al., the use of beta-blockers resulted in 64% decrease in SCAD recurrences over a median of 3.1 years [17].

##### Statins

Since SCAD in not due to plaque rupture, statins prescription is controversial: cohort studies have shown disparate results for the use of statins in the prevention of SCAD recurrences [17].

### 3.7. SCAD Complications

SCAD complications include propagation of dissection and recurrent myocardial infarction due to dissection of another coronary artery. The incidence of in-hospital recurrent MI is 5–10% [17,30] and the risk of extension of dissection in medically treated patients is 17% over a period of 14 days [32]. Clinical symptoms suspected for complication occurrence include recurrent or worsening angina, EKG dynamic modifications, cardiac enzymes elevation.

Among patients readmitted to the hospital within the first month, 45% of them experience recurrent MI, frequently occurring within 2 days after discharge [5]. Therefore, a hospitalization of 3–5 days is warranted to detect SCAD complications [46,47].

### 3.8. SCAD Recurrence

SCAD-associated mortality is low (1% over 3 years), while SCAD recurrence is high, with 17–18% of patients experiencing recurrent MI over 3 to 4 years [17]. The majority of these events are coronary dissections, recurrent SCAD being defined as new-onset coronary dissections unrelated to the index event, usually involving a different coronary vessel [47]. Conversely, SCAD extension is defined as the propagation of a known intramural hematoma causing relapsing symptoms, dynamic EKG alterations, or a new increase of cardiac enzymes. The rate of recurrences varies from 5 to 15% over a median period of 22 to 27 months [30,35]. Clinical factors predisposing to SCAD recurrence include arterial hypertension [17], fibromuscular dysplasia, migraine [3], and coronary artery tortuosity [53].

Beta-blockers should be the first-choice drug therapy, having demonstrated their beneficial effect on preventing recurrence [17].

Whether pregnancy is associated with SCAD recurrences is unknown, since this association has been reported only in case series [54]. However, since SCAD is a prevalent cause of MI in pregnant women and pregnancy-related SCAD has a more severe course, counselling for women pregnancy seeking should be warranted.

### 3.9. Assessment of Extracardiac Vascular Abnormalities

Adjunctive total body vascular imaging tests including CT-scan and/or MRI should be recommended after a SCAD to investigate extracardiac arterial abnormalities, which have a high prevalence in this patient’s subset. Conversely, the beneficial effect of periodic vascular examinations is unknown.

## 4. Practical Considerations

Over the years, a reduction in the number of urgent revascularizations with avoidance of CABG and reduction in PCIs in favour of SCAD medical treatment, has been progressively reported in literature. However, percutaneous coronary intervention still plays a role when SCAD involves major proximal coronary vessels presenting with STEMI and severe hemodynamic compromise.

Medical therapy, with the availability of more potent P2Y12 antiplatelet agents, has gained a pivotal role in the treatment of SCAD, especially in case of NSTE-ACS presentations, as confirmed by our experience and published data.

The condition poses diagnostic challenges and significant therapeutic dilemmas given the small number of case series not allowing for statistically significant conclusions and the lack of research to guide management.

## 5. Conclusions

Ongoing challenges in SCAD cover several aspects including: a prompt and accurate diagnosis together with improving outcomes; uncertainty about management of associated conditions, risk stratification and prevention of complications and recurrences; recommendation for physical activity, reproductive planning and genetic evaluation; finally, lack of high-quality evidence for acute and long-term management (mostly retrospective and observational data) [55].

## Figures and Tables

**Table 1:** Monzino’s SCAD dataset (January 2007–November 2021).

	*n* = 54
Age, years, median (IQR)	56 (49, 69.75)
**Gender**	
Male, *n* (%)	6 (11.1%)
Female, *n* (%)	48 (88.9%)
**Cardiovascular risk factors**	
None, *n* (%)	1 (1.8%)
Hypertension, *n* (%)	12 (22%)
Smoking habit, *n* (%)	6 (11.1%)
Dyslipidemia, *n* (%)	8 (14.8%)
Family history of CAD, *n* (%)	6 (11.1%)
Diabetes mellitus, *n* (%)	1 (1.8%)
**Previous medical history**	
Negative, *n* (%)	35 (64.8%)
Myocardial infarction, *n* (%)	8 (14.8%)
Previous PCI, *n* (%)	6 (11.1%)
Previous cancer, *n* (%)	1 (1.8%)
Anemia, *n* (%)	1 (1.8%)
Peripheral artery disease, *n* (%)	1 (1.8%)
Previous SCAD, *n* (%)	8 (14.8%)
**Clinical presentation**	
NSTEMI-ACS, *n* (%)	34 (62.9%)
Emergency	20 (37.1%)
Anterior STEMI, *n* (%)	10 (18.5%)
Inferior STEMI, *n* (%)	6 (11.1%)
Lateral STEMI, *n* (%)	4 (7.5%)
Critical state at presentation	1 (1.8%)
Cardiac arrest, *n* (%)	0
Cardiogenic shock, *n* (%)	1 (1.8%)
Pulmonary edema, *n* (%)	0
**Treatment**	
Medical therapy, *n* (%)	40 (74%)
PCI, *n* (%)	13 (24.2%)
CABG, *n* (%)	1 (1.8%)

IQR: interquartile range; CAD: coronary artery disease; SCAD: Spontaneous coronary artery dissection; NSTEMI-ACS: non-ST elevation acute coronary syndrome, STEMI: ST-elevation acute myocardial infarction; PCI: percutaneous coronary intervention, CABG: coronary artery bypass graft.

**Table 2:** Literature case series compared to Monzino’s data.

Authors	Type of Study	No. of Patients	Mean Age	Female	Clinical Presentation		Treatment			
					STEMI	NSTEMI	Medical Therapy	PCI	CABG	Thrombolytic Therapy
Nakashimi et al. [8]	Retrospective	63	46 (±10)	59 (93.6%)	55 (87.3%)	8 (12.7%)	28 (44.4%)	34 (53.9%)	1 (1.5%)	0
Saw et al. [19]	Retrospective	168	52.1 (±9.2)	155 (92.3%)	44 (26.2%)	124 (73.8%)	131 (77.9%)	30 (17.9%)	5 (2.9%)	2 (1.2%)
Tweet et al. [35]	Retrospective	189	44 (±9)	174 (92.1%)	37 (19.6%)	151 (79.9%)	94 (49.7%)	89 (47.1%)	6 (3.2%)	0
Rogowski et al. [36]	Prospective	64	53 (±11.2)	60 (93.7%)	19 (29.7%)	44 (68.7%)	56 (87.5%)	7 (10.9%)	1 (1.6%)	0
Mortensen et al. [37]	Retrospective	22	48.7 (±8.9)	17 (77.2%)	16 (72.7%)	4 (18.1%)	7 (31.8%)	13 (59%)	2 (9.1%)	0
Vanzetto et al. [38]	Retrospective	23	46 (±9)	17 (73.9%)	7 (30.4%)	14 (60.8%)	10 (43.5%)	11 (47.8%)	2 (8.6%)	0
Hiroki et al. [39]	Retrospective	23	45 (±11)	23 (100%)	11 (47.8%)	12 (52.1%)	11 (47.8%)	4 (17.4%)	6 (26.1%)	2 (0.9%)
Rashid et al. [40]	Retrospective	21	53.3 (±8.8)	20 (95.2%)	8 (38.1%)	13 (61.9%)	15 (71.4%)	6 (28.6%)	0	0
Alfonso et al. [41]	Prospective	45	53 (±11)	23 (51.1%)	14 (31.1%)	9 (20%)	12 (26.7%)	15 (33.3 %)	0	0
Boulmpou et al. [42]	Retrospective	9	56 (±11)	8 (88.8%)	0	9 (100%)	9 (100%)	0	0	0
Roura et al. [43]	Prospective	34	47 (±12)	32 (94.1%)	19 (55.9%)	15 (44.1%)	26 (76.5%)	8 (23.5%)	0	0
Abreu et al. [44]	Retrospective	27	56 (±11)	22 (81.5%)	10 (37%)	15 (55.5%)	12 (44.4%)	15 (55.5%)	0	0
Centro Cardiologico Monzino (Milan)	Retrospective	54	59.2 (18.4)	48 (88.9%)	20 (37%)	34 (63%)	40 (74.1%)	13 (24.1%)	1 (1.8%)	0

## Data Availability

Dataset has been uploaded and published in Zenodo https://zenodo.org/record/5759074.
